# Genetic Drivers of Progression in Alzheimer's Disease

**DOI:** 10.1002/alz70861_108400

**Published:** 2025-12-23

**Authors:** Shane Fernandez, Celeste E Cohen, Ahmad R Ehyaei, Umran Yaman, Tenielle Porter, Dervis Salih, John A. Hardy, Simon M. Laws, Maryam Shoai, The Australian Imaging, Biomarker and Lifestyle (AIBL) Flagship Study of Ageing

**Affiliations:** ^1^ Centre for Precision Health, Edith Cowan University, Joondalup, Western Australia Australia; ^2^ Collaborative Genomics and Translation Group, School of Medical and Health Sciences, Edith Cowan University, Joondalup, Western Australia Australia; ^3^ Wellcome Sanger Institute, Cambridge UK; ^4^ Max‐Planck‐Institute for Intelligent Systems, Tubingen Germany; ^5^ University College London, Institute of Neurology, London UK; ^6^ UK Dementia Research Institute, University College London, London UK; ^7^ Department of Neurodegenerative Disease, UCL Queen Square Institute of Neurology, London UK; ^8^ Reta Lila Weston Institute of Neurological Studies, London UK; ^9^ Collaborative Genomics and Translation Group, Edith Cowan University, Joondalup, Western Australia Australia

## Abstract

**Background:**

Elucidating the genetic determinants influencing Alzheimer's disease (AD) progression is crucial for the rational design of new targeted and efficacious therapeutic interventions.

**Method:**

We conducted a genome‐wide study utilizing data from 382 participants with amyloid beta status, genetic profiles, and longitudinal clinical assessments, sourced from the Alzheimer's Disease Neuroimaging Initiative and the Australian Imaging, Biomarkers and Lifestyle Flagship Study of Ageing. Mixed effects models were employed to evaluate the rate of change in Mini‐Mental State Examination scores as a quantitative measure of disease progression

**Results:**

Variants on chromosome 22q12.1 mapped to ZNRF3, MN1, PIPNB genes reached genome‐wide significance and together with nominally significant loci (*p* <5×10−6) highlighted a role for neuronal resilience (both excitatory and inhibitory neurones) and anti‐viral immune responses. The APOE locus was not significantly associated with the rate of progression. Among the known AD risk genes, the *OAS* locus achieved nominal significance suggesting the interferon pathway has a role in determining rates of progression.

**Conclusion:**

The absence of a significant association between APOE and AD progression suggests that alternative pathological pathways, beyond amyloid beta, may represent viable targets for impeding disease advancement. These pathways appear to involve neuronal resilience and immune system modulation.

